# A novel combined technology for treating hypertrophic scars: adipose tissue extract combined with fractional CO_2_ laser

**DOI:** 10.3389/fphys.2023.1284312

**Published:** 2023-10-30

**Authors:** Yuan Cai, Jiao Tian, Jianyi Li, Xing Liu, Fang Li, Lanfang Zhang, Shune Xiao, Changsha Jia, Chengliang Deng

**Affiliations:** ^1^ Department of Dermatology, Affiliated Hospital of Zunyi Medical University, Zunyi, Guizhou, China; ^2^ Department of Surgery, Zunyi Medical College, Zunyi, Guizhou, China; ^3^ Department of Clinical Medicine, Zunyi Medical College, Zunyi, Guizhou, China; ^4^ Department of Burns and Plastic Surgery, Nanxishan Hospital of Guangxi Zhuang Autonomous Region, Guilin, Guangxi, China; ^5^ Department of Burns and Plastic Surgery, Afliated Hospital of Zunyi Medical University, Zunyi, Guizhou, China; ^6^ Department of Dermatology, Guiyang First People’s Hospital, Guiyang, Guizhou, China

**Keywords:** scar treatment, adipose tissue extract, fractional Co_2_ laser, hypertrophic scars, adipogenesis

## Abstract

**Introduction:** Owing to the need for liposuction and its unsuitability for allogeneic transplantation, the clinical application of stromal vascular fraction gel (SVF-gel) combined with fractional CO_2_ laser for scar treatment is limited. Adipose tissue extract (ATE), rich in cytokines and growth factors, offers a more convenient option for clinical practice as it can be easily prepared using purely physical methods and has low immunogenicity. We aimed to evaluate the effectiveness of ATE combined with fractional CO_2_ laser in the treatment of hypertrophic scars.

**Methods:** ATE was prepared using discarded liposuction fluid from patients undergoing liposuction. A rabbit ear hypertrophic scar model was established and treated with ATE, fractional CO_2_ laser, or a combination. PBS was used as a control. The scar appearance and histological changes were observed. The immunohistochemistry method was used to evaluate the expression of α-SMA, while perilipin was detected using immunofluorescence. Additionally, the level of adipogenic signal C/EBPα and PPARγ mRNA was studied.

**Results:** Following treatment, the volume of hypertrophic scar decreased, resulting in a softer texture and thinner dermis. Additionally, there was a decrease in the infiltration of inflammatory cells, and the collagen arrangement became looser and more regular, and the expression of α-SMA also decreased, with the combination of ATE and fractional laser showing the most significant improvement. Moreover, the combination group was found to promote subcutaneous fat regeneration and increase the expression of adipogenic signals C/EBPα and PPARγ.

**Conclusion:** The combination of ATE and fractional CO_2_ laser treatment has been shown to inhibit the development of hypertrophic scars. This effect may be attributed to the enhancement of adipogenesis and decrease in collagen deposition.

## 1 Introduction

Hypertrophic scars can cause distressing symptoms such as itching, pain, and potential damage to one’s appearance. In severe cases, they may lead to local functional limitations or loss, resulting in sleep disturbances and psychological disorders (Finnerty et al., 2016). Therefore, scarring significantly affects patients’ quality of life. While a variety of treatment options are available for hypertrophic scars, a universally accepted standard treatment does not currently exist. Recent studies have demonstrated the effectiveness of autologous fat grafting and laser therapy in improving the aesthetic and functional outcomes of scar treatment. These interventions have been found to enhance joint mobility, alleviate symptoms of discomfort such as pain and itching, and promote scar healing (Klinger et al., 2013; Huang et al., 2015; Lei et al., 2017; Klinger et al., 2020). However, the clinical application of autologous fat grafts may be limited owing to the release of oil droplets from adipocyte necrosis, which can exacerbate the local inflammatory response. Moreover, the concentration of adipose-derived stem cells (ASCs) in adipose tissue is typically low (Spiekman et al., 2017). These factors should be carefully evaluated when considering the potential use of autologous fat in medical procedures.

In our previous study, we explored the application of a stromal vascular fraction gel (SVF-gel), which contains a high concentration of ASCs, in conjunction with fractional CO_2_ laser therapy to treat hypertrophic scars (Xiao et al., 2023). The animal experiments demonstrated that this combined treatment greatly improved the aesthetic appearance of scars. Histological analysis revealed clearer skin structure, more organized collagen fibers, and visible new fat cells after treatment. Clinical experiments further indicated significant reductions in pain and itching in patients with hypertrophic scars after treatment. In addition, the appearance and function of the treated areas showed significant improvements. These findings suggest the potential clinical applicability of this combined treatment for hypertrophic scars. However, the clinical application of SVF-gel is limited owing to the requirement of liposuction during each application, low patient acceptance, inability to be used for allogeneic applications, and limited commercial promotion opportunities.

Adipose tissue extract (ATE) (Yu et al., 2018; Deng et al., 2019) is a rich source of cytokines and extracellular vesicles that can be obtained through purely physical methods. Unlike other techniques, ATE does not require cell separation or *in vitro* culture, making it simpler and less expensive. Moreover, ATE eliminates the risks of immunogenicity and tumorigenicity since it does not contain cells. Researchers have applied ATE to various conditions, such as photoaging (Deng et al., 2019), ischemic diseases (Yu et al., 2018), and wound healing (Na et al., 2017), demonstrating promising therapeutic effects. Based on our analysis, it appears that ATE may offer simpler promotion and clinical application than SVF-gel. Therefore, we speculate that ATE may have a therapeutic effect similar to that of SVF-gel in the treatment of hypertrophic scars. To investigate the effectiveness of treating hypertrophic scars, we utilized a combination of ATE and fractional CO_2_ laser. Our findings indicate that intralesional transplantation of ATE following laser treatment results in an improvement in scar appearance, and we studied the potential therapeutic mechanism.

## 2 Materials and methods

### 2.1 Animals and ethics

Sixteen adult New Zealand white rabbits, weighing 2.5 ± 0.2 kg, were housed in a single cage (Chongqing Kangge Biotechnology Co., Ltd, Chongqing, China). All experimental procedures were approved by the Animal Experimental Ethics Committee of the Affiliated Hospital of Zunyi Medical University and were carried out in accordance with the guidelines of the National Health and Medical Research Committee (China) [KLLY (A)-2020-015].

### 2.2 Hypertrophic scar model of rabbit ear

A bilateral ventral hypertrophic scar model of rabbit ears was developed following a previously described procedure (Xiao et al., 2023). In short, the rabbits were anesthetized with an intraperitoneal injection of 30 mg/kg pentobarbital. After routine disinfection, a rectangular wound measuring 5.5 cm × 1.5 cm was created on the ventral side of each ear, and the skin and perichondrium were removed. After 4 weeks, red, hard hypertrophic scars protruding from the skin surface had formed.

### 2.3 Preparation of ATE

Waste adipose tissue from patients undergoing liposuction in the Department of Plastic Surgery of the affiliated Hospital of Zunyi Medical University was collected, and ATE was prepared according to a previously described procedure (Yao et al., 2017; Yu et al., 2018; He et al., 2019). The collected adipose tissue was rinsed with physiological saline to remove red blood cells, then centrifuged at 1200 × g for 3 min. After centrifugation, the upper and lower layers of liquid were discarded and the middle fat layer was collected. Mechanical emulsification was conducted by repeatedly moving the middle fat layer back and forth 30 times between two 10-cm^3^ syringes. The syringes were connected using a female-to-female Luer-Lock connector with an inner diameter of 1.4 mm. The emulsified fat was frozen at −80°C and thawed at 37°C to further destroy adipose tissue. After one cycle of freezing and thawing, the sample was centrifuged again at 1200 × g for 5 min. Following centrifugation, the fat separates into four layers. The top layer contains oil droplets, the second layer consists of unbroken fat, and the fourth layer contains fragments, all of which are discarded. The liquid from the third layer, ATE, was collected. The final extract was obtained by sterilizing and removing cell debris using a 0.22 μm filter. Finally, the extract was divided into smaller portions and frozen for subsequent use.

### 2.4 Intervention of rabbit ear hypertrophic scar model

After successfully establishing the rabbit ear hypertrophic scar model, 32 scars were randomly assigned to four groups to eliminate subsequent experimental differences: ATE plus laser (*n* = 8), ATE (*n* = 8), laser (*n* = 8), and saline (*n* = 8) groups. The treatment parameters of the fractional CO_2_ laser (AP, United States) were as follows: Deep Fx, energy: 25 mJ, shape: 2, size: 10, pulse: 1, and density: 5%. The injection volume of ATE was 0.1 mL/cm^2^.

### 2.5 Observation of rabbit ear hypertrophic scar

Digital photographs of the scars were taken at 30, 60, and 90 days; the color, thickness, texture, and size of the scars were observed. Scars were also assessed using the Vancouver Scar Scale (VSS) (Nedelec et al., 2000) as shown in Table 1, which includes the assessment of vascularity, pliability, pigmentation, and height. The full-thickness scar samples measuring 5.5 cm × 1.5 cm were collected 30 and 90 days after treatment. Half of the collected tissue was fixed with paraformaldehyde for histological staining, while the other half was frozen at −80°C for follow-up experiments.

**TABLE 1:** Vancouver scar scale used in our study.

Vascularity		Pliability		Pigmentation		Height	
Characteristic	Score	Characteristic	Score	Characteristic	Score	Characteristic	Score
Normal	0	Normal	0	Normal	0	Flat	0
Pink	1	Supple	1	Hypopigmentation	1	<2 mm	1
Red	2	Yielding	2	Mixed	2	2–5 mm	2
Purple	3	Firm	3	Hyperpigmentation	3	>5 mm	3
		Ropes	4				
		Contracture	5				

^a^ Including assessment of vascularity, pliability, pigmentation, and height.

### 2.6 Histological analysis

The sections were regularly dewaxed and rehydrated. Hematoxylin-eosin staining (HE) and Masson’s trichrome were performed following the manufacturer’s instructions to evaluate the histological changes of the scar. In each group, five slices were randomly chosen for HE staining. Using ImageJ software, the vertical distance from the highest point of scar tissue to the cartilage surface (A) and the vertical distance from the surrounding normal skin surface to the cartilage surface (B) were measured. The scar hyperplasia index (scar Index = A/B) was calculated, where a higher index indicates a more prominent superficial scar. Similarly, for Masson’s trichrome staining, five slices were randomly selected in each group, and the collagen density was quantified using Image J software.

### 2.7 Immunohistochemistry

The sections were routinely dewaxed and rehydrated. They were then washed with PBS and blocked with 3% H_2_O_2_ enzyme for 10 min to remove endogenous peroxidase. They were then washed 3 times with PBS for 3 min. To perform antigen retrieval, the tissue is soaked in EDTA repair solution, then rinsed with PBS 3 times for 3 min each. The tissue is then sealed with goat serum at room temperature for 30 min. Next, a circle was drawn on the surrounding tissue using an oil-based pen. The primary antibody (α-SMA; 1:60; Abcam, Cambridge, MA, United States) is dropped onto the tissue and incubated overnight at 4°C. Next, the HRP-labeled secondary antibody was added dropwise and incubated at 37°C for 30 min, DAB chromogenic solution was added dropwise, and dehydrated. After hematoxylin counterstaining, the transparent sections were sealed with neutral gum. At least five fields were observed under a microscope in each section, and the positive α-SMA expression was counted using ImageJ software.

### 2.8 Immunofluorescence analysis

After routine dewaxing and rehydration, the sections were incubated overnight with guinea pig anti-rabbit perilipin (1:400, Progen, Germany), followed by incubation with secondary antibodies (goat anti-guinea pig IgG-488; 1:200, Thermo Fisher Scientific, Cambridge, MA) for co-staining. The nuclei were stained with DAPI staining solution (1:200; Sigma, St. Louis, MO). Images were obtained using a confocal laser scanning microscope, and perilipin protein was quantitatively detected using ImageJ.

### 2.9 Quantitative reverse-transcription PCR

Quantitative reverse-transcription PCR (qRT-PCR) was performed according to standard procedures. The fold change of each target gene was normalized to the fold change of GAPDH mRNA. The primer sequences used were as follows:

C/EBPα: Forward ACAACAGGCCAGGTTTCC

Reverse TCCCCGTGTCCTCCTATC

PPARγ: Forward GAGCAAAGAAGTCGCCAT

Reverse CTG​GTC​GTT​CAA​GTC​AAG​G

GAPDH: Forward TGT​GGC​CGA​GGA​CTT​TGA​TT

Reverse TAC​ACA​AAT​GCG​ATG​CTG​CC

### 2.10 Statistical analysis

The results were analyzed using SPSS 20.0, and the mean ± standard deviation was used to express the statistical results. After performing a normality test, differences among the four groups were compared using one-way analysis of variance (ANOVA). The Bonferroni test was used for homogeneous variances, while Dunnett’s t3 test was used for non-homogeneous variances. A *p*-value less than 0.05 was considered statistically significant.

## 3 Results

### 3.1 Inhibition of hypertrophic scar by ATE combined with fractional CO_2_ laser

**FIGURE 1:**
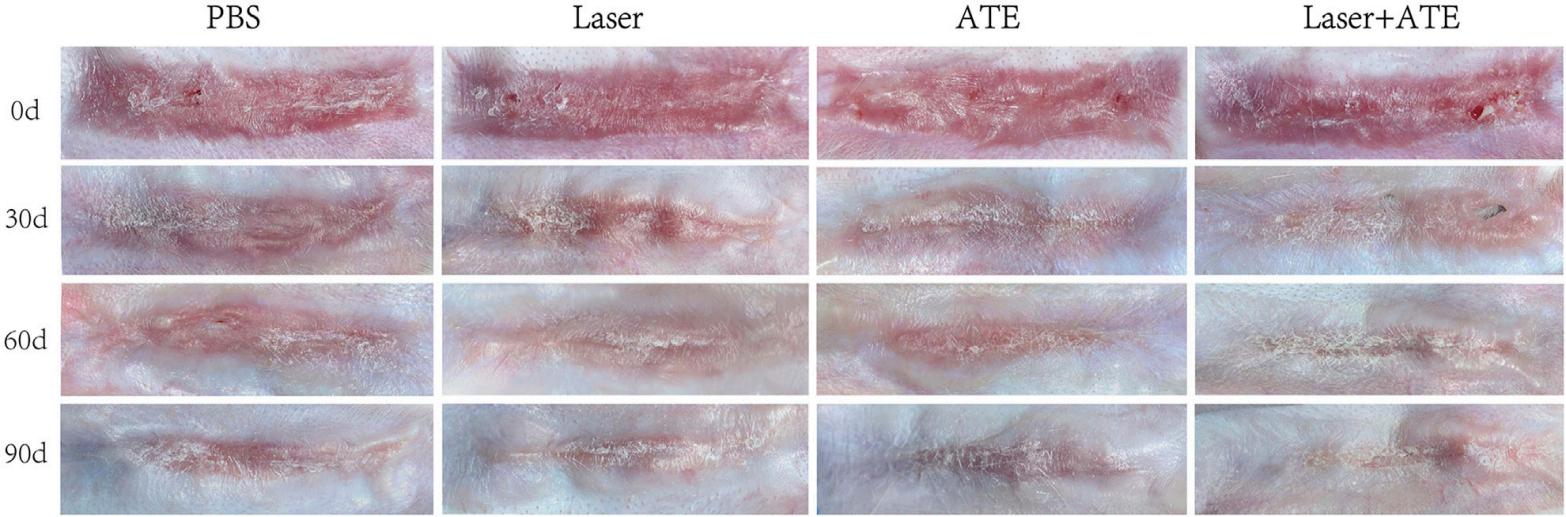
General observation of hypertrophic scar after treatment with PBS, ATE, laser and ATE+laser. After 90 days, the color, thickness and texture of the scar in the combined group were closer to the normal skin, and the degree of improvement was the most obvious, followed by the ATE and laser group, while the stiff and protruding scar could still be seen in the control group.

Before the intervention, all rabbits' wounds had been completely epithelialized, and hyperplastic scars with a red bulge and hard texture were observed. The hyperplastic area did not exceed the edge of the original wound. During the intervention, all animals remained well without skin wound infection or ulcer formation. Over time, the color of the scars in each group gradually faded, and those treated with laser or ATE gradually flattened, narrowed, and softened. At each time point, the combination group showed the best effect, followed by the ATE and laser groups. Notably, on the 90th day, the scars in the combination group almost disappeared and resembled normal skin (Figure 1).

The Vancouver Scar Scale (VSS) results were consistent (Table 2). The VSS scores of each group at each time point after the intervention were lower than those before the intervention, and this difference was significant (*p* < 0.0001). As the intervention time was prolonged, the VSS scores of each group continued to decrease. The combination group showed the most significant decrease, followed by the ATE group and the laser group. The differences in VSS scores among the groups were also significant (*p* < 0.05).

**TABLE 2:** Comparison of VSS score at different time points before and after intervention in each group (x ± s).

Group	Before intervention (*n* = 4)	After intervention		
		30 d	60 d	90 d
PBS (n = 4)	10.50 ± 0.93*	9.25 ± 1.58^c^ ^e^ ^f^	7.87 ± 1.13^c^ ^e^	6.37 ± 1.41^c^ ^e^
Laser (n = 4)		7.75 ± 0.71^b^	7.13 ± 0.83^b^	5.50 ± 1.31^b^
ATE (n = 4)		6.75 ± 1.03	6.25 ± 0.71^a^	4.25 ± 1.28^a^
ATE + Laser (n = 4)		5.63 ± 1.19	4.75 ± 0.71	2.75 ± 1.39
*F*	-	23.780	47.643	42.077
*P*	-	0.000	0.000	0.000

*The results showed that there were differences in each group before intervention and in each time point, p < 0.05.

^a^ represents the difference between the combination group and the ATE, group at each time point, *p* < 0.05.

^b^ represents the difference between the combination group and the Laser group at each time point, *p* < 0.05.

^c^ represents the difference between the combination group and the PBS, group at each time point, *p* < 0.05.

^d^ represents the difference between the ATE, group and the Laser group at each time point, *p* < 0.05.

^e^ represents the difference between the ATE, group and the PBS, group at each time point, *p* < 0.05.

^f^ represents the difference between Laser group and PBS, group at each time point, *p* < 0.05.

### 3.2 ATE plus fractional CO_2_ laser improves histological structure of hypertrophic scar

**FIGURE 2:**
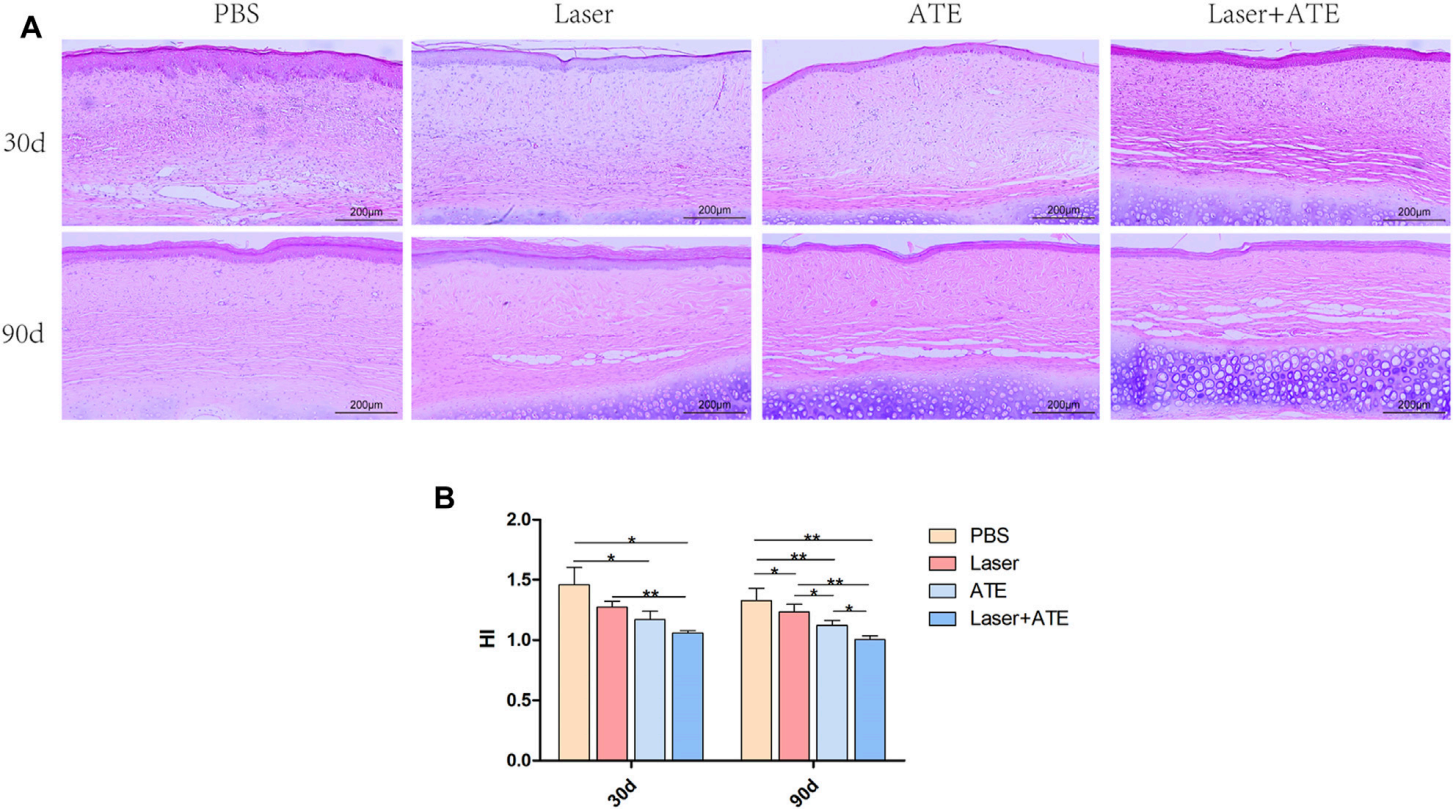
Histopathological evaluation of ATE combined with CO_2_ laser in the treatment of hypertrophic scar. **(A)** HE staining showed that the dermis and epidermis became thinner and the infiltration of inflammatory cells decreased after treatment, especially in the combination group, but almost no improvement in the PBS group. **(B)** The scar hyperplasia index showed that the scar hyperplasia index decreased after treatment, especially in the combination group. Scale bar = 200 μm. (*p < 0.05, **p < 0.01).

HE staining was performed on the scar samples at 30 and 90 days after the intervention to observe the histological morphology of the scar (Figure 2A). The results revealed that on the 30th day, the scarred dermis and epidermis in the PBS group exhibited significant thickening accompanied by a substantial infiltration of inflammatory cells. Conversely, the combination group, ATE group, and laser group showed a thinner epidermis and dermis than the PBS group, and the infiltration of inflammatory cells was observed to be reduced. On the 90th day, the thickness of the dermis and epidermis of the scars in each group decreased, and there was a significant reduction in inflammatory cells. Notably, the combination group showed the most significant improvement in dermal thickness and clearer skin layers, which aligns with the general pictures.

The scar hyperplasia index (Figure 2B) 30 days after the intervention decreased in the combination group, ATE group, and laser group compared to that in the PBS group. After 90 days, the scar hyperplasia index further decreased in all groups, with significant differences observed between the four groups. The combination group and laser group showed more noticeable differences than the PBS group, as did the ATE group compared to the PBS group (*p* < 0.01). Additionally, significant differences in the scar hyperplasia index were found between the combination group and ATE group, ATE group and laser group, and laser group and PBS group (*p* < 0.05).

Importantly, at 90 days after the intervention, adipocytes were visible in the scar dermis of the combination group, ATE group, and laser group, with the combination group showing a more abundant presence. Scar improvement appeared to be related to the number of adipocytes in the scar.

### 3.3 ATE plus fractional CO_2_ laser promotes collagen remodeling of hypertrophic scars

**FIGURE 3:**
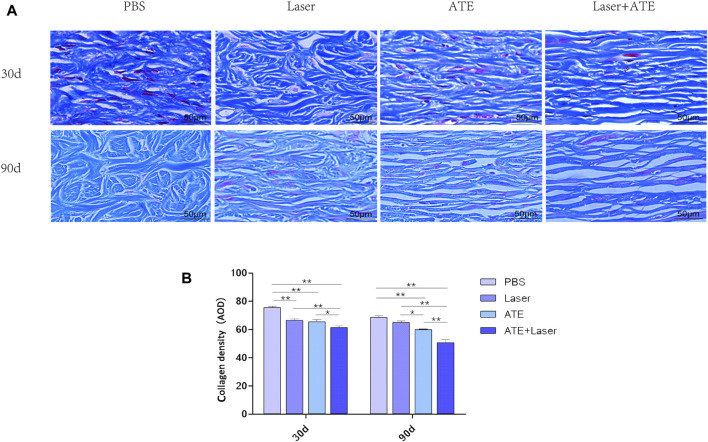
ATE combined with CO_2_ laser can reduce collagen deposition in hypertrophic scar. **(A)** Masson staining showed that the arrangement of collagen fibers in the combination group was the loosest and regular, while the collagen fibers in the PBS group were disordered and dense. **(B)**. The quantitative results of collagen expression in each group. Scale bar = 50 μm. (*p < 0.05, **p < 0.01).

Masson’s trichrome staining was performed on scar specimens at 30 and 90 days after the intervention to evaluate collagen deposition (Figure 3A). On the 30th day after the intervention, the reduction of collagen deposition in each group was not significant. Dense and disordered collagen fibers were observed in the PBS group and the laser group, while the collagen fibers in the combination group and the ATE group appeared loose and neat. On the 90th day, collagen deposition decreased in all groups except the PBS group, and the collagen arrangement became more consistent. In the combination group, the collagen gaps were widened and the collagen fibers were the loosest and most regular. In the PBS group, dense and irregular collagen was still visible.

Collagen quantitative analysis (Figure 3B) revealed that at 30 days, the collagen density in the PBS group was significantly higher than that in the laser and ATE groups. The combination group had the lowest density of collagen, which was significant (*p* < 0.01). At 90 days, the collagen density in all groups was lower than that at 30 days. The combination group had the lowest collagen expression, followed by the ATE group, laser group, and PBS group, respectively, with a significant difference compared to each group (*p* < 0.05).

### 3.4 ATE plus fractional CO_2_ laser relieves scar fibrosis

**FIGURE 4:**
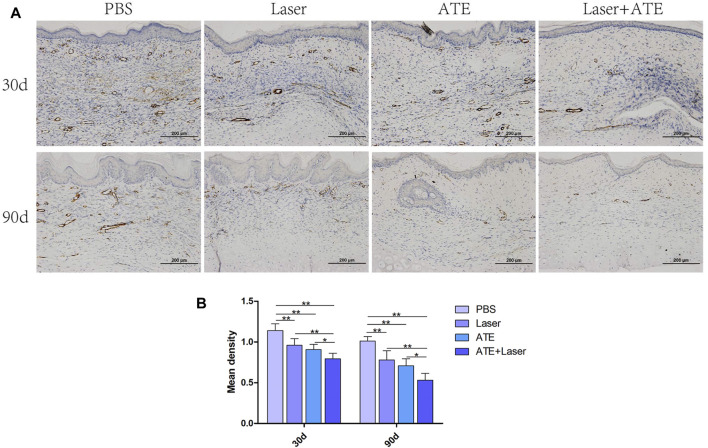
Immunohistochemical staining of α-SMA. **(A)** The results showed that the positive staining area of the combined group was the least. **(B)** The quantitative expression of α-SMA. Scale bar = 200 μm. (*p < 0.05, **p < 0.01).

Because α-SMA plays an important role in scar fibrosis, we performed immunohistochemical staining of α-SMA in scars treated with ATE and laser (Figure 4). At 30 days after treatment, the PBS group exhibited a large number of brown areas and the expression of α-SMA was noticeably higher in comparison to the other three groups. By the 90th day, the brown staining area in the sections of each group decreased further than that by the 30th day, and the expression of α-SMA showed a downward trend, especially in the combination group (*p* < 0.05), indicating that the effect of combined therapy was better than that of single treatment.

### 3.5 ATE and fractional CO_2_ laser promote adipogenesis in scar

**FIGURE 5:**
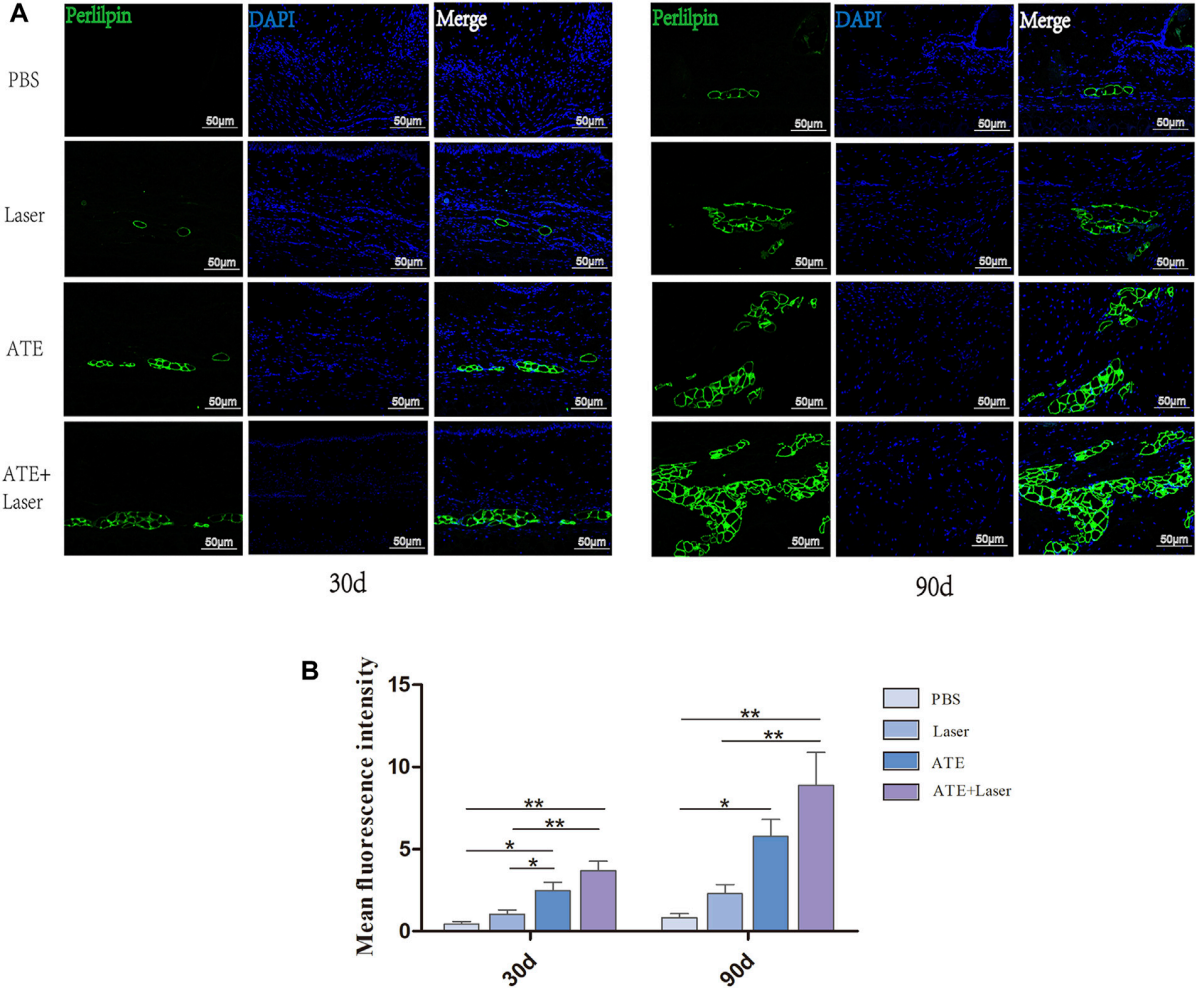
The immunofluorescence staining of perilipin showed fat regeneration in hypertrophic scar. **(A)** The results showed that the expression of perilipin in the combined group was significantly higher than that in the other three groups, especially 90 days after intervention, followed by ATE group, while the expression was not obvious in laser group and PBS group. **(B)** The quantitative expression of perilipin. Perilipin, green represents fat cells; DAPI, blue represents the nucleus; Scale bar = 50 μm (*p < 0.05, **p < 0.01).

The aforementioned studies showed that treatment with ATE plus laser resulted in a tendency of normal scar skin structure, intact epidermis, thin dermis, and abundant subcutaneous adipose tissue. The results of histological staining showed that the scar treated with ATE plus laser had a satisfactory effect on ECM remodeling and subcutaneous adipose tissue regeneration. Therefore, to validate the regeneration of subcutaneous fat in scar tissue, we performed perilipin immunofluorescence staining on paraffin sections at days 30 and 90 after treatment (Figure 5). As shown in the figure, green fluorescent protein was observed in the combination group and ATE group on the 30th day, with significantly higher expression than that in the laser group and PBS group. On the 90th day, the expression of green fluorescent protein was elevated in all groups compared to that on the 30th day, especially in the combination group, while minimal expression was observed in the PBS group (*p* < 0.05). Although no significant difference was observed between the combination and ATE groups at the two time points, perilipin expression was higher in the combination group than in the ATE group.

**FIGURE 6:**
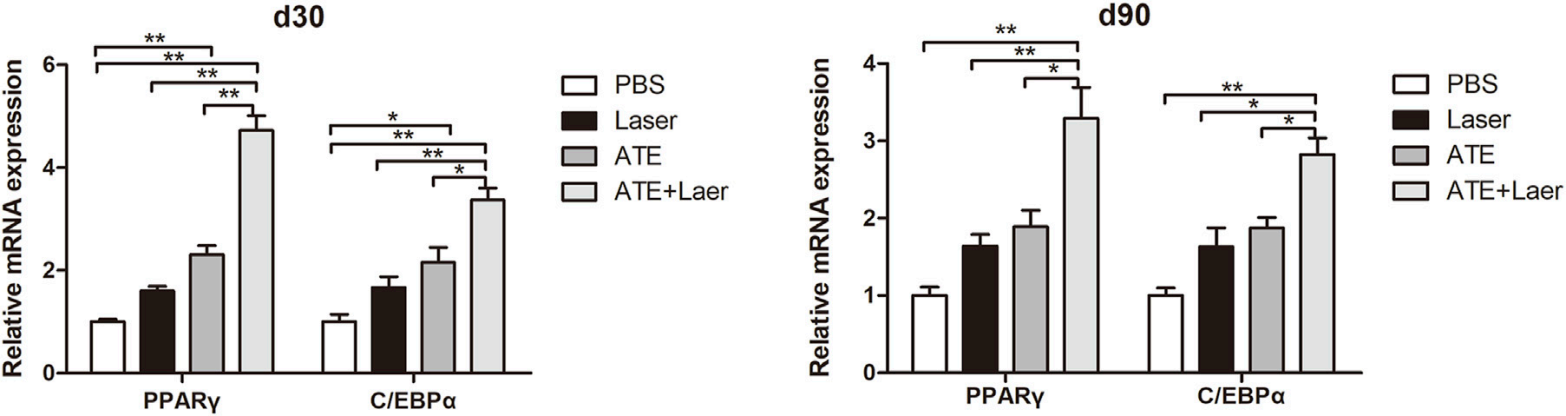
Expression of adipogenic signaling markers C/EBPα and PPARɤ mRNA.The results of qPCR showed that the expression of C/EBPα and PPARɤ mRNA in the combination group was significantly increased no matter 30d or 90d after the intervention, followed by the ATE group and the laser group, and the PBS group had the lowest expression. (*p < 0.05, **p < 0.01).

To further explore the effect of ATE combined with fractional CO_2_ laser on adipogenesis in scar tissue, we used qPCR to measure the expression of adipogenic markers C/EBPα and PPARγ mRNA (Figure 6). The results revealed a significant increase in the expression of C/EBPα and PPARγ mRNA in the combination group after treatment, followed by the ATE group and the laser group, with the lowest expression observed in the PBS group (*p* < 0.05).

## 4 Discussion

In addition to energy storage, adipocytes play crucial roles in regulating metabolism and immunity, as well as promoting wound repair (Kruglikov and Scherer, 2016; Zhang et al., 2019). Studies conducted in mouse models have demonstrated the beneficial effects of adipocytes in wound healing. For instance, the use of a biological dressing containing adipocytes has been shown to promote full-thickness excisional wound repair (Morissette Martin et al., 2015). Furthermore, the ablation of dermal adipocytes in transgenic mice has been found to delay skin wound healing (Shook et al., 2020). The regeneration of skin appendages during wound healing is also accompanied by the emergence of mature adipocytes (Plikus et al., 2017). Further research has revealed that adipocytes have the ability to regulate the biological functions of fibroblasts during wound healing, thereby playing a significant role in the overall wound healing process (Schmidt and Horsley, 2013). In addition to their role in wound healing, adipocytes have also been found to play a significant role in fibrotic diseases in recent years. The loss of adipose tissue is commonly observed in various human diseases and experimental animal models, and it is often associated with pathological tissue fibrosis. A recent study investigated the lesional skin of patients with systemic sclerosis (SSc) and animals with experimentally induced fibrosis. The findings revealed a reduction in intradermal adipose tissue located beneath the reticular dermis. This reduction was accompanied by visible adipocyte atrophy and malformation, and the affected adipose tissue was surrounded by fibrillar collagen (Marangoni et al., 2015). In addition to SSc, this phenomenon has also been reported in cases of LMNA-related hereditary laminopathy and other mutations associated with generalized and familial partial lipodystrophy (Bereziat et al., 2011). Adipose tissue loss is also associated with fibrosis in acquired lipodystrophies that are secondary to panniculitis, autoimmune diseases, restrictive dermopathy, scarring alopecia, anorexia, and cancer cachexia, as well as antiviral therapy with protease inhibitors (Garg, 2004; Bing and Trayhurn, 2009; Karnik et al., 2009; Rivera-Gonzalez et al., 2014).

As a result, adipose tissue has become a subject of increasing research interest. Wang et al. also revealed significant regeneration of subcutaneous adipose tissue following the local transplantation of SVF-gel for hypertrophic scar treatment (Wang et al., 2019). Xiao et al. used an SVF-gel combined with a fractional CO_2_ laser to treat hypertrophic scars (Xiao et al., 2023). This combined approach demonstrated a synergistic effect, resulting in significant improvements in scar structure, collagen remodeling, and adipose tissue regeneration within the scar. The expression of adipogenesis-related markers C/EBPα and PPARγ were also found to be highly expressed, suggesting the involvement of adipogenesis in scar treatment.

While SVF-gel shows promise, it has limitations in clinical application owing to the requirement for liposuction during each transplantation and restrictions on allotransplantation and commercial use. Recent studies suggest that ASCs primarily exert their biological role through paracrine growth factors and cytokines, which are closely related to regeneration and metabolism (Cai et al., 2020). As a result, ATE, which is abundant in growth factors and cytokines, can be obtained through purely physical methods without cell isolation or *in vitro* culture and may serve as a preferred alternative to ASCs therapy.

Our previous animal experiments demonstrated that ATE-treated mice exhibited faster wound healing rates and significantly increased blood vessel density compared to the control group (He et al., 2019). In addition, ATE can induce adipogenesis *in vitro* (Sarkanen et al., 2012; He et al., 2019). When used as a cell culture supplement at a concentration above 200 mg/mL, it effectively promotes triglyceride accumulation in human adipose stem cells within a week and upregulates the expression of PPARγ, a marker of adipoblast differentiation. Several studies have also shown (Lu et al., 2016) that ATE significantly enhances adipose tissue regeneration in the adipose tissue engineering compartment model compared to that in the PBS control group. In this model, ATE group displays improved morphology and structure of the adipose flap, a thinner capsule, increased blood vessel formation, and significantly higher expression of angiogenic growth factor and adipose formation markers such as C/EBPα and PPARγ. These findings suggest that cytokines and growth factors present in ATE create a favorable microenvironment for adipose tissue formation.

Based on these observations, we conducted a study using a rabbit ear hypertrophic scar model to evaluate the effects of ATE and fractional CO_2_ laser, both alone and in combination, for the treatment of hypertrophic scars. The results showed that the combined treatment had a more pronounced improvement in scar appearance than ATE or fractional CO_2_ laser alone, as evidenced by a decrease in the VSS score. Histological analysis demonstrated a reduction in the elevation index of the scar, looser and more regular collagen fibers, decreased expression of α-SMA, and enhanced regeneration of adipocytes. Furthermore, the expression of adipogenic markers C/EBPα and PPARγ was increased. The enhanced effect of the combination group may be attributed to collagen remodeling caused by the precise thermal damage zone generated by the fractional CO_2_ laser, coupled with the adipogenesis-inducing factors present in ATE that promote adipocyte regeneration within the scar. These results further highlight adipogenesis as one of the mechanisms involved in the treatment of hypertrophic scars.

Excessive deposition of extracellular matrix is an important feature in all types of tissue fibrosis. As important cells in tissue fibrosis, myofibroblasts have a strong ability to contract, synthesize, and secrete collagen and matrix. When there is an imbalance in their synthesis and secretion capacity, pathological scars form (Zhang et al., 2020). The expression of α-SMA represents a characteristic feature of fibroblasts-to-myofibroblasts transformation (Venugopal et al., 2022), and reflects the secretion level of myofibroblasts. In our study, Masson staining and α-SMA immunohistochemical staining were conducted, revealing that the combination group exhibited the least collagen deposition, a more regular and orderly collagen arrangement, broader gaps, and the expression of α-SMA decreased. These findings were consistent, whether at 30 days or 90 days. By contrast, the PBS group exhibited disordered and dense collagen fibers, with the highest expression of α-SMA. These differences were significant, and the results are consistent with those in previous studies. For example, El-Zawahry et al. used fractional laser to treat burn scars and found that the collagen density in the scar dermis of patients decreased, new collagen in scar tissue replaced irregular collagen, proportion of normal collagen increased, and arrangement became more regular (El-Zawahry et al., 2015). In addition, Lee et al. used a CO fractional laser to treat burn scars and found that the number of fibroblasts in the scar dermis decreased (Lee et al., 2016). ATE, which is rich in a variety of growth factors and cytokines, may be an important factor for scar improvement. Therefore, these results show that ATE and fractional CO_2_ laser have a synergistic effect, and a combined treatment can significantly improve the appearance and structure of scar tissue. However, it should be noted that this study focuses on surface mechanisms and further studies are needed to elucidate the underlying molecular pathways involved.

**FIGURE 7:**
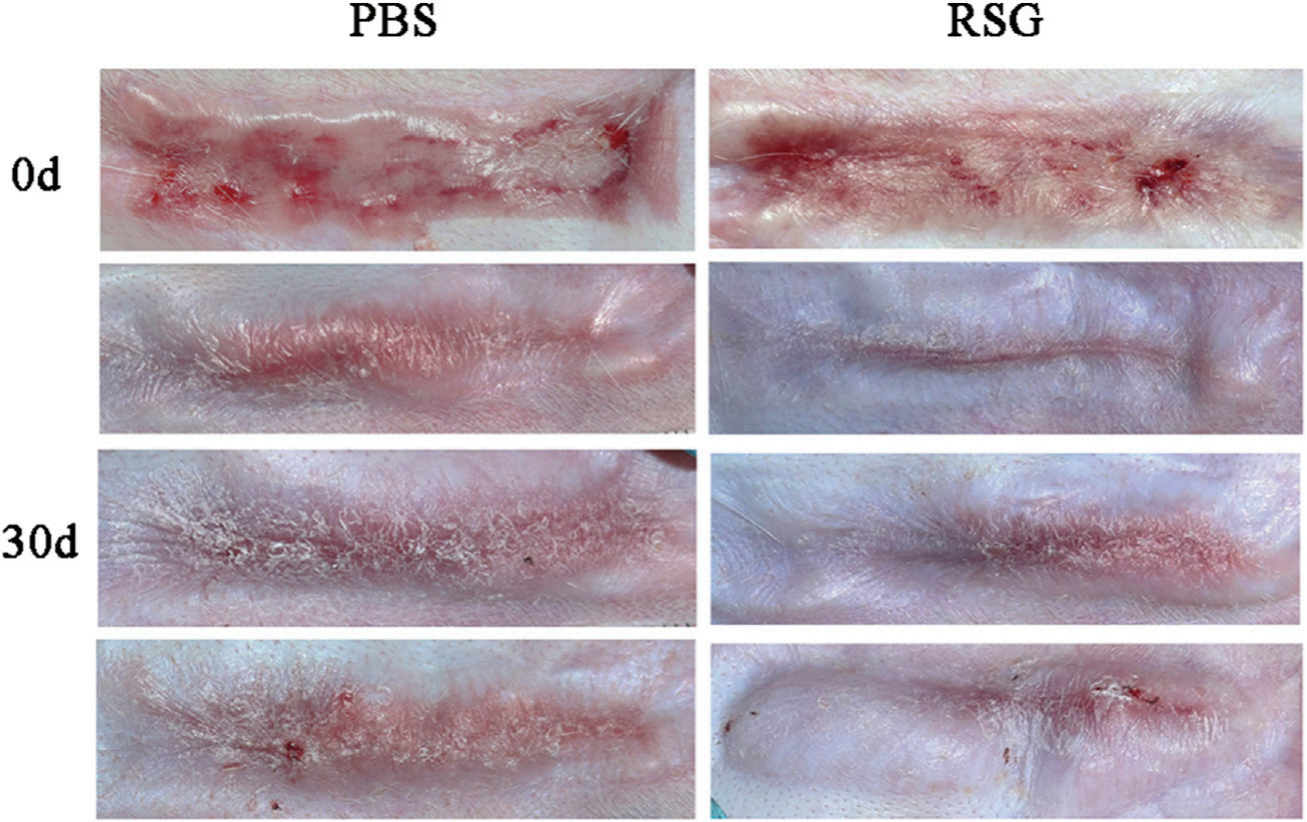
Gross observation of hypertrophic scars after treatment with PBS and Rosiglitazone.The results showed that compared with the PBS group, the improvement in scar color, thickness and texture was more obvious after rosiglitazone treatment.

Recent studies have shown that PPARγ has an anti-fibrosis effect by weakening or even inhibiting the expression of transforming growth factor-β (Zhang et al., 2009; Deng et al., 2012; Vetuschi et al., 2018). Decreased PPARγ expression and enhanced TGF-β signal are associated with progressive fibrosis (Wei et al., 2010). Our study also found that the higher the expression of PPARγ, the less collagen deposition and α-SMA expression, and the more pronounced the improvement in scar appearance. Therefore, to determine whether the new adipocytes in the scar play an antifibrotic effect through PPARγ, we re-established the rabbit ear hypertrophic scar model and intervened with rosiglitazone, a PPARγ agonist. Images of the animals (Figure 7) showed that 30 days after the intervention, the scar in the rosiglitazone group became thinner, lighter in color, and softer in texture than that in the PBS group.

However, this study has limitations as the source of new adipocytes in the scar is not clearly identified. Plikus et al. reported that BMP-2 and BMP-4 secreted by hair follicles reprogrammed myofibroblasts into adipoblast lines during wound healing in mice (Plikus et al., 2017). Another study by Hoserst demonstrated the activation of adipogenic signals C/EBPβ and PPARγ in co-cultured myofibroblasts, hypertrophic scar, and keloid fibroblasts treated with BMP-4 or adipocyte-CM, although no lipid droplets were observed (Hoerst et al., 2019). These findings suggest that newborn adipocytes are derived from fibroblasts in scars. However, our study also observed a few adipocytes in scars after laser treatment, raising the need for further investigation into whether these new adipocytes originate from remaining stem cells or fibroblasts within the scar and whether the results of this study are solely phenomenological. While our study suggests that intra-scar adipogenesis may be one of the mechanisms for the treatment of hypertrophic scars, it only provides a superficial understanding. The mechanism of ATE combined with lattice laser in the treatment of hypertrophic scars has not been extensively studied, which is also a limitation of this study. Furthermore, it is important to compare the effectiveness of ATE in combination with fractional CO_2_ laser and adipose tissue. Considering that adipose tissue is rich in ASC and ATE contains various cytokines secreted by adipose tissue, it is possible that adipose tissue may be more effective in treating hypertrophic scars than ATE. However, it must be noted that liposuction is necessary for each application of adipose tissue, which leads to low patient acceptance. On the other hand, the ATE is non-immunogenic and can be frozen and used multiple times in one liposuction extraction. Therefore, from a clinical perspective, the non-immunogenic ATE may be more easily promoted. All in all, further research is required to elucidate the specific mechanism of action of this treatment in addressing hypertrophic scars. Additionally, it is important to verify and compare the efficacy of this treatment method with alternative approaches, such as adipose tissue, ASC and SVF-gel etc.

In conclusion, the combination of ATE and fractional CO_2_ laser can effectively improve hypertrophic scars. The observed mechanism of action may be related to the induction of adipogenesis and extracellular matrix remodeling. While further research is required to fully understand the underlying processes, this therapeutic approach not only provides basic research data for the clinical application of ATE and lasers in the treatment of scars but also provides a new adipogenic acellular therapy for the clinical management of hypertrophic scars.

## Data Availability

The original contributions presented in the study are included in the article/Supplementary Material, further inquiries can be directed to the corresponding authors.
